# The spatial structure of correlations in natural scenes shapes neural coding in mouse primary visual cortex

**DOI:** 10.1186/1471-2202-15-S1-P36

**Published:** 2014-07-21

**Authors:** Rajeev V Rikhye, Mriganka Sur

**Affiliations:** 1 Department of Brain and Cognitive Sciences, Massachusetts Institute of Technology, Cambridge, MA 02139, USA

## 

**Figure 1:**
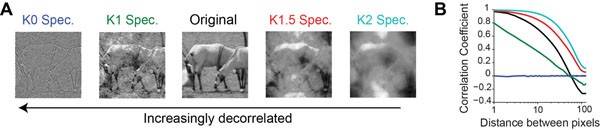
**Selectively perturbing spatial correlations alters the appearance of natural movies. **(**A**) Example frames from a natural movie with 4 different levels of correlation. (**B**) Autocorrelation function quantifying the spatial correlations within the frames shown in (**A**). All colors are labeled in (**A**).

Natural scenes, although complex in appearance, contain numerous statistical regularities. For instance, owing to surfaces and textures, neighboring pixels in natural images are strongly correlated in both space and time (Figure 1B). These correlations create strong dependencies between neurons that may limit neural coding efficiency. Thus, to produce an efficient representation, it has been proposed that neural circuits in the early visual system function to remove extraneous stimulus correlations. Although extensively studied in the retina and LGN, the mechanisms of efficient coding in primary visual cortex (V1) remain elusive. The goal of this study is to establish a relationship between population coding in V1 and spatial correlations in natural scenes.

Mathematically, the Fourier amplitude spectrum describes second-order correlations between pixels. In contrast, the phase spectrum describes higher-order correlations, which arise due to edges and other salient features. To understand how stimulus correlations influence coding in V1, we developed a novel technique that allowed us to systematically perturb the Fourier amplitude spectrum of natural movies, while keeping the edge structure intact. Using this method, we generated movies with four different levels of spatial correlation (Figure 1A). Visually, edges in the most decorrelated movies (K0 spectrum) appear shaper, while edges in the most strongly correlated movies appear occluded (K2 spectrum). To assess how populations of neurons respond to these movies, we performed high-speed 2-photon calcium imaging in layer 2/3 of mouse V1. This method permitted us to monitor the activity of close to 100 neurons with high spatial and temporal resolution.

We hypothesized that neuronal networks in V1 should decorrelate strongly correlated movies. Interestingly, we found that neurons responded to strongly correlated movies (K1.5, K2) with higher firing rates and with less variability than the decorrelated movies (K0, K1). Response reliability was computed as the average over all pairwise correlations between all single trial responses to one given movie. This analysis revealed that response reliability decreased monotonically with increasing levels of spatial decorrelation in the movie (arrow in Figure 1A). Because the edge structure of these movies was not altered, this result suggests that computations performed by V1 neurons are modulated by spatial correlations. Next, we investigated how interactions between neural assemblies are altered by stimulus correlations. We found that signal correlation between neurons (SC) decreased monotonically with increasing level decorrelation. Noise correlation (NC), however, remained unchanged by the perturbation. By plotting SC as a function of separation between neurons, we discovered that strongly correlated movies evoked long-range correlations between neurons. On the other hand, spatially decorrelated stimuli resulted in weak and separation-invariant correlations. In other words, the structure of SC between V1 neurons is very closely matched to the structure of spatial correlations in the stimulus.

In summary, our work demonstrates that neurons in mouse V1 adapt their coding strategy to match spatial correlations in the visual stimulus. Thus, unlike the retina where stimulus redundancy is removed via whitening, V1 neurons preserve stimulus correlations. This strategy *improves* coding efficiency as it allows neurons to learn about the structure of the environment.

